# Combined Effect of HPV and Several Gene SNPs in Laryngeal Cancer

**DOI:** 10.3390/medicina56020081

**Published:** 2020-02-17

**Authors:** Aušra Stumbrytė-Kaminskienė, Živilė Gudlevičienė, Daiva Dabkevičienė, Irina Mackevičienė

**Affiliations:** 1 Biobank, National Cancer Institute, P. Baublio 3b, LT-08406 Vilnius, Lithuania; zivile.gudleviciene@nvi.lt; 2 Laboratory of Clinical Oncology, National Cancer Institute, P. Baublio 3b, LT-08406 Vilnius, Lithuania; daiva.dabkeviciene@nvi.lt; 3 Department of Head and Neck Surgery and Oncology, National Cancer Institute, Santariškių 1, LT-08660 Vilnius, Lithuania; irina.mackeviciene@nvi.lt

**Keywords:** laryngeal squamous cell carcinoma, single nucleotide polymorphisms, patient survival, human papillomavirus, multivariate analysis

## Abstract

*Background and objectives:* Laryngeal squamous cell carcinoma (LSCC) is one of the most common head and neck tumors. The molecular mechanism of LSCC remains unclear. The aim of this study was to evaluate the prevalence of Human papillomavirus (HPV) and single nucleotide polymorphisms (SNPs) of *TP53, MDM2, MDM4, MTHFR, CASP8,* and *CCR5* genes in LSCC, and to assess their correlations with patient survival. *Materials and Methods:* 49 LSCC patients were enrolled in this study. PCR and qRT-PCR were used to detect, identify, and quantify HPV. SNPs were genotyped using PCR and PCR-RFLP. *Results:* By analyzing the interactions of the SNPs of the genes with clinical parameters, the majority of patients with lymph node status (N1,2) were identified as carriers of *MDM2* *T/G*, *CASP8* *ins/del*, *CCR5* *wt/wt* SNP. Cluster analysis showed that patients with *MDM2* *T/T* SNP survive longer than patients identified as *CASP8* *ins/ins*, *MTHFR* *C/C,* and *MDM4* *A/A* variant carriers; meanwhile, LSCC patients with *MDM2* *T/T* polymorphic variant had the best survival. Multivariate analysis showed that HPV-positive patients without metastasis in regional lymph nodes (N0) and harboring *CASP8* *ins/del* variant had the best survival. Meanwhile, HPV-negative patients with identified metastasis in lymph nodes (N1 and N2) and *CASP8* *ins/del* variant had poor survival. *Conclusions:* This finding suggests patients survival prognosis and tumor behavior are different according HPV status, SNP variants, and clinical characteristics of the LSCC.

## 1. Introduction

A common malignancy found in the head and neck tumor is laryngeal squamous cell carcinoma (LSCC) [1,2], with 151,000 new cases diagnosed worldwide [3] each year and 90,000 associated deaths [4,5]. LSCC usually affects men over 50–60 years of age [6] and is strongly related with tobacco smoking and alcohol use [7]. Meanwhile, some infectious pathogens such as HPV may act as associated carcinogenic factors [8]. Smoking trends each year are decreasing. Nevertheless, LSCC cases were found to be increasing among young people with an increasing spread of HPV infection [9]. HPV infection has been shown to cause oropharyngeal cancer. The association between HPV and LSCC is based on the morphological similarities between the cervical and the squamous epithelium, as well as the detection of the commonest oncogenic HPV genotypes (HPV-16, HPV-18) in both cervical and laryngeal cancers (LC) [10,11]. However, the clinical significance of HPV infection remains to be determined. HPV belongs to the *Papillomaviridae* family and infects squamous cells and mucous membranes of humans. Prevalence of HPV in LC patients varies between 3 and 85% [12]. HPV has been shown to play a crucial role in the molecular pathways through its viral oncoproteins E6 and E7 [13]. However, not only chemical factors (tobacco, alcohol) or viral infection plays and important role in the LSCC development. Other factors, the genetic predisposition and susceptibility to the HPV infection could play a strengthening role in the carcinogenesis. Multiple biologically relevant SNPs may have more accurate predictive power of cancer prognosis. Moreover, the functional SNPs in genes *TP53* (rs1042522), *MDM2* (rs2279744), *MDM4* (rs4245739), *MTHFR* (rs1801133), *CASP8* (rs3834129), and *CCR5* (rs333) combination, could affect survival of LSCC patients [14]. The p53 protein functions as the ‘tumor suppressor’ by regulating the cell cycle to conserve genomic stability and prevent mutation [15]. The SNP rs1042522 is Arginine (Arg) to Proline (Pro) amino acid substitution in position 72 of the p53 protein. Arg form is more efficient in apoptosis induction, whereas the Pro form induces more G1 arrest and is better at activating p53 dependent DNA repair [16]. Murine double-minute 2 (MDM2) oncoprotein plays an important role as a negative regulator p53 [17,18]. The cellular MDM2 protein acts as an E3 ubiquitin-ligase by transferring ubiquitin onto p53, thereby targeting it to proteasome-mediated degradation, uninitiated growth arrest or apoptosis of infected cells [19]. The SNP 309 T/G has been shown to be associated with increased risk of cancer [18]. Cells with heterozygous allele have a higher level of *MDM2* and a lower apoptotic response than cells with *T/T* [20]. In humans, *MDM2* SNP 309 is shown to be associated with accelerated tumor formation in both hereditary and sporadic cancers [17,21].

The MDM family includes *MDM2* and *MDM4* genes—a key negative regulator of p53 [17,22]. Mdm4 binds to the p53 transactivation domain and inhibits transcriptional activity, and thus contributes to tumor formation [22]. Research shows that the *MDM4* rs4245739 has been associated with overall cancer risk [23]. Folate is important in deoxynucleoside synthesis to provide methyl groups and in intracellular methylation reactions. Methylenetetrahydrofolate reductase (MTHFR) is an important enzyme in folate metabolism [24]. The C677T SNP in *MTHFR* (rs1801133), which regulates the release of active folate in the body, may have reduced activity [25,26]. Epidemiological evidence suggests that the SNPs encoding the enzymes involved in folate metabolism may increase the risk of head and neck cell carcinoma (HNSCC) by altering DNA methylation synthesis and genomic stability [25]. Given that folate participates in intracellular pathways, it seems plausible that patients with HNSCC may respond differently to treatments, based on genetic SNP [25,26].

Caspases are the main regulative and executive enzymes in the apoptosis pathway. Caspase 8 (CASP8) are most important proteins of the caspase family [27]. The efficiency of apoptosis in an organism may be the consequence of *CASP8* SNPs. The *CASP8*-652 6N *ins/del* (rs3834129) SNP has been shown toinfluence the progression of several cancers [28]. Del allele and *ins/del* genotype of the *CASP8* gene may play a protective role in carcinogenesis [29]. Chemokines are chemoattractant proteins of low molecular weight that promote adhesiveness of target cells [30]. SNPs in this gene, in particular the Δ32 mutation (a 32 bp deletion in the *CCR5* gene), leads to the synthesis of non-functional protein [30,31,32].

## 2. Materials and Methods

In the period from September of 2013 to December of 2014, 49 patients (45 males and 4 females) from the Head and Neck Surgery and Oncology Department of the National Cancer Institute (Vilnius, Lithuania), with diagnosis of primary LSCC, were invited to participate in the scientific study. Study protocol was initiated by the Vilnius Regional Committee for the Biomedical Research (6 November 2013 permission No. 158200-13-638-204). Patients were included into the study according to the following criteria: all of them had primary LSCC and none of them had received therapy prior to surgery. All LSCC patients signed Informed consent before the surgery. After surgery, LSCC was confirmed by an experienced pathologist in the National Center of Pathology (Vilnius, Lithuania) for all of the patients included in our study. The remaining operating material after the diagnosis was collected in the Biobank at the National Cancer Institute and stored till the scientific research. Before the experiment, all samples were analyzed for the presence of HPV. After that, the SPN analysis of various genes was performed.

**Figure 1:**
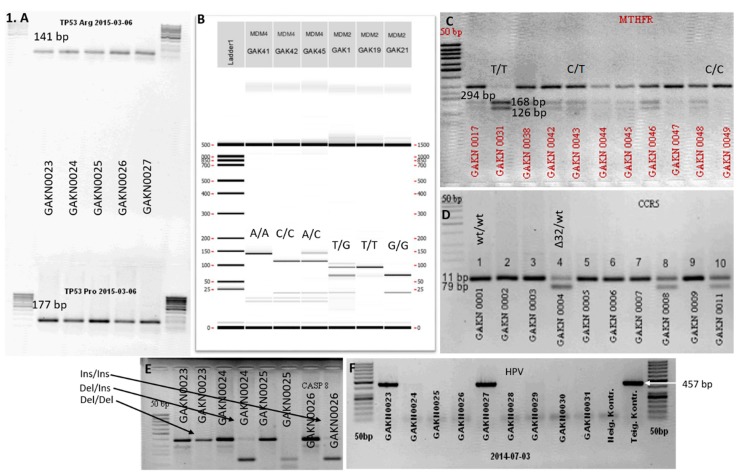
*TP53, MDM2, MDM4, MTHFR, CASP8,* and *CCR5* genes polymorphic variants of laryngeal tumor tissue using agarose electrophoresis and LabChip GX/GX II Touch capillary electrophoresis gel: (**A**)**—***TP53* polymorphic variants (141 bp—Arg; 177 bp—Pro); (**B**)—*MDM4* and *MDM2* gene polymorphic variants of laryngeal tumor tissue using LabChip GX/GX II Touch capillary electrophoresis method. Single nucleotide polymorphisms (SNPs) of gene *MDM2* (89 bp—*G/G*; 64 bp, 25 bp—*T/T*; 89 bp, 64 bp, 25 bp—*T/G*); SNPs of gene *MDM4* (132bp—*A/A*, 132 bp, 111 bp—*A/C*, 111 bp—*C/C*); (**C**)—SNPs of gene *MTHFR* (168 pb, 126 bp—*T/T*; 294 bp—*C/C*; 294 bp, 168 bp, 126 bp—*C/T*); (**D**)—SNPs of gene *CCR5* (79 bp—Δ*32/*Δ*32*; 111 bp—*wt/wt*; 111 bp, 79 bp—*wt/*Δ*32*); (**E**)—SNPs of gene *CASP8* (396 bp, 291 bp—*del/del*; 396 bp, 291 bp, 139 bp—*del/ins*; 396 bp, 139 bp—*ins/ins*); (**F**)—HPV (457 bp).

DNA was purified by an organic extraction method according to the approved standard operating procedure (SOP) at the Biobank of the National Cancer Institute. From each tumor sample, extracted DNA was used for PCR analysis of HPV positivity detection and SNP investigation of *TP53* (rs1042522), *CASP8* (rs3834129) and *CCR5* (rs333) genes (Figure 1A,D–F). After PCR reactions with specific primers (Table 1), for product visualization the gel electrophoresis method was used to identify HPV presence in the samples and all studied SNPs.

Later phylogenetic group (PG) of HPV, viral copy number, and the total number of copies of the virus in cells were determined by qPCR. Amplification was carried out using the Rotor-Gene Q amplificatory and Rotor-Gene Q software (version 2.1.9.9). *AmpliSens^®^ HPV HCR screen-titre-FRT PCR* kit was composed of PCR-mix-1-FRT HPV A9, A7 and A5/A6 phylogenetic groups according HPV *E1-E2* gene-based primers. Endogenic control with β–globin was performed for each PCR cycle. The number of copies of the virus in the cell was calculated using the formula: log (HPV DNA copies/human DNA copies) × 200,000 = log (HPV of 100,000 cells). Finally, an exact genotyping of HPV was performed using multiplex PCR and *Seegene HPV6 ACE Genotyping* kit. Additionally, *MDM2* (rs2279744), *MDM4* (rs4245739) and *MTHFR* (rs1801133) genotypes were determined by PCR—restriction fragment length polymorphism (RFLP). An 89 bp fragment covering the *MDM2* gene, was first amplified with specific primers. Then PCR products were digested with restriction enzyme TaqI at 65 °C for 8 min. The T/G genotype was identified by the existence of 89 bp, 64 bp and 25 bp fragments. The PCR products of gene *MDM4* were digested with restriction enzyme MspI at 37 °C for 8 min. SNPs were identified by the existence of 134 bp, 111 bp and 23 bp fragments. Finally, the PCR products of gene *MTHFR* (rs1801133) were digested with restriction enzyme Hinfl at 37 °C for 8 min. and the results of this study were fragments of 294 bp, 168 bp, 126 bp. All fragments have been investigated by LabChip GX I Touch capillary electrophoresis and electrophoresis in agarose gel method (Figure 1B,C). 

Statistical comparisons among groups were performed by the Chi-square test. Survival was analysed by Kaplan-Meier comparison using both log-rank test for two survival curves or Gehan-Breslow test for multiple survival curves, and with multivariate Cox proportional harard analysis. Calculation of sample size for Cox proportional harard analysis was performed using R package ‘powerSurvEpi’. *p* value of <0.05 was considered statistically significant. Statistical analysis was performed using SigmaPlot 12.3 and STATISTICA 10.0 software, if not stated otherwise. 

## 3. Results

### 3.1. Association of SNPs Distribution and Clinical-Pathological Characteristics in LSCC Patients

Clinical parameters were evaluated for a group of 49 LSCC patients. Median patients age—63 year (IQR = 14). According to the tumor stage and lymph node status at the time of diagnosis, the distribution of patients was as follows: 3 cases (6.1%) were T1, 10 cases (20.4%) were T2, 19 cases (38.8%) were T3, and 17 cases (34.7%) were T4, while 33 cases (67.4%) were N0, 7 cases (14.2%) were N1, and 9 cases (18.4%) were N2.

For all patients’ status of HPV and the *TP53*, *MDM2*, *MDM4*, *MTHFR* (rs1801133), *CASP8* (rs3834129) and *CCR5* (rs333), polymorphism were investigated. For the evaluation of SNP interactions with clinical parameters, HPV infection and survival rates, the frequencies of the examined genes SNPs were calculated.

After HPV detection, viral infection was found in 42.86% (21 out of 49) of all LSCC cases. Viral genotyping showed, that HPV 16 was the dominant type (17 out of 21 cases, 80.95%) in the HPV-positive tumor samples; in several cases HPV 18 type (3 out of 21 cases, 14.9%) was detected and in one case double infection was observed. After HPV phenotyping, the phylogenetic group A9 was detected in all patient samples but one case of HPV A9. After testing the number of HPV copies in the laryngeal tissues, the highest number of copies was 4.49 lg HPV copy/cell and the minimum value was 0.05 lg HPV copy/cell. Using SNP analysis, the most common polymorphic genes variants identified in our study were as follows: *MDM2* gene position 309 *T/G* (53.1%), *MDM4* 1q 32 *A/A* (67.3%), *MTHFR* gene position 667 *C/T* (48.9%), *CASP8* 652 *ins/del* (49.0%), and *CCR5*-Δ32 *wt/wt* (79.6%) polymorphic variant. In the case of *TP53* Arg72Pro SNP, only the heterozygous polymorphic variant of Arg/Pro has been identified for all patients (Table 2).

By analyzing the correlation of the SNPs of the genes with clinical parameters (TNM classification) established the relationship between the presence of *MDM2*, *CASP8*, *CCR5* genes and region lymph nodes (N). The majority of patients with N1–2 were also identified as carriers of *MDM2* *T/G*, *CASP8* *ins/del*, *CCR5* *wt/wt* SNPs (*p* = 0.24; *p* = 0.19; *p* = 0.11). In the SNP analysis, polymorphic variants *C/T* and *C/C* (*p* = 0.22) of gene *MTHFR* was usually determined in T4 stage tumors. The detection of *TP53*, *MDM2*, *MDM4*, *MTHFR*, *CASP8,* and *CCR5* genes SNPs has been designed to find links to HPV infection and clinical-pathological characteristics. HPV-positive and HPV-negative patients failed to determine the reliable difference between the frequencies of tested SNPs. However, we found that polymorphic variants of the *MDM2* gene *G/G* (4 out of 28, 18.8%) were more common in HPV-negative samples compared with HPV-positive. Differences in the frequency distribution of *CASP8* and *CCR5* genes in the HPV-positive and HPV-negative groups were also observed (*p* = 0.22; *p* = 0.16). *CASP8* *del/del* and *CCR5* *wt/*Δ*32* alleles were more commonly detected for HPV-positive patients: 7 cases out of 28 (25.00%) and 8 of 28 cases (28.60%), respectively. For 33 patients, regional lymph node (N0) metastatsis were not detected, for 16 patients regional metastasis was stated (N1–N2).

### 3.2. Survival Analysis according to TP53c.215 G > C (Arg72Pro), MDM2c.-5 + 309 G > T, MDM4c.1q32 A > C, MTHFRc.677 C > T, CASP8c.-652 6N ins/del, CCR5c.-Δ32 Genes SNPs

The main task of our study was to determine the relationship between polymorphic variants of genes *TP53*c.215 G > C (Arg72Pro), *MDM2*c.-5 + 309 G > T, *MDM4*c.1q32 A > C, *MTHFR*c.677 C > T, *CASP8*c.-652 6N *ins/del, CCR5*-Δ32, HPV infection and patients’ survival. Survival was estimated by the Kaplan-Meier method.

**Figure 2:**
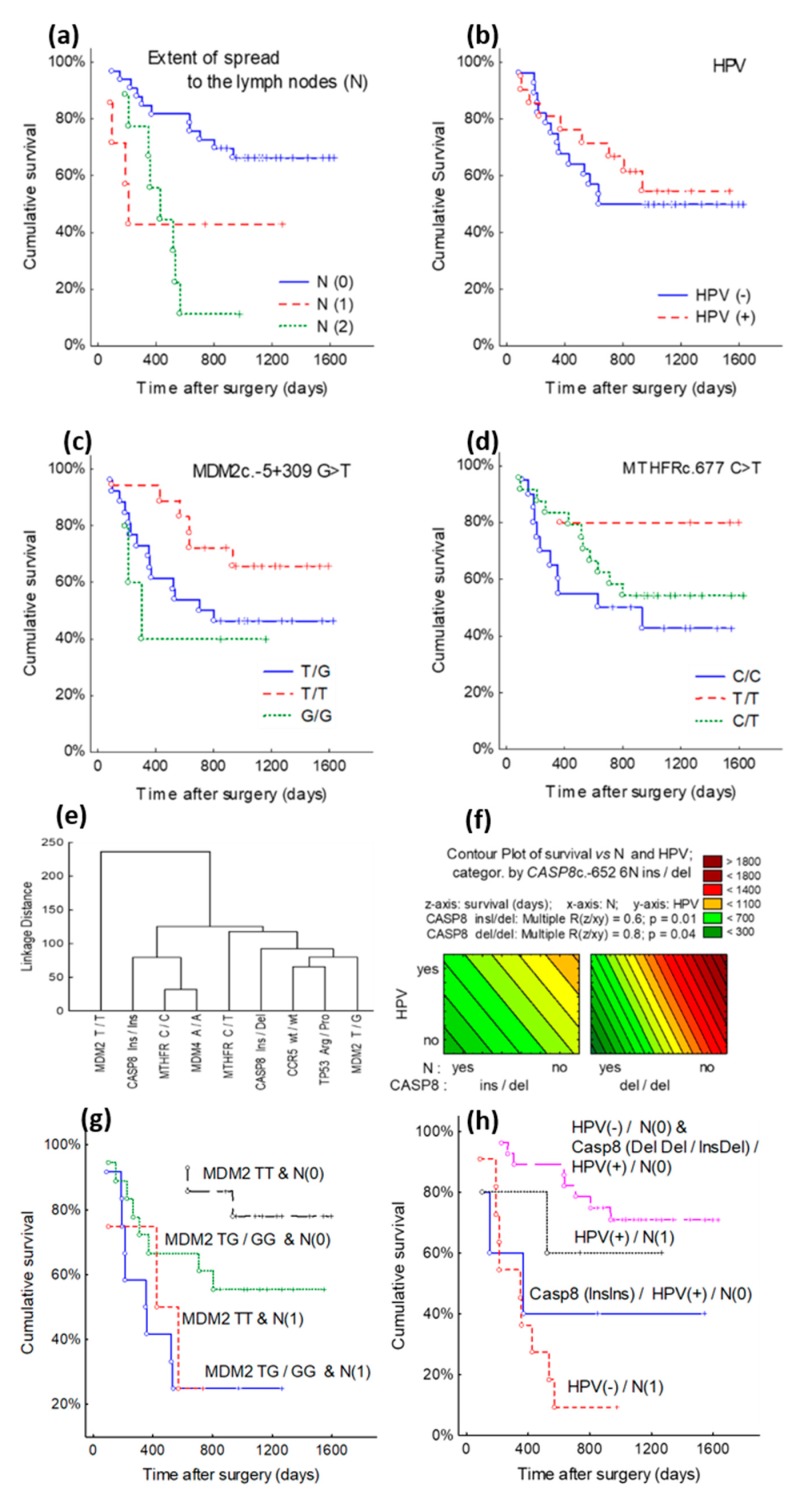
Cancer patient survival curves according to genes SNPs, Lymph Node and human papiiloma virus (HPV): Patients’ survival rates: (**a**) Lymph Node (N0-2) (*p* = 0.01); (**b**) HPV+/- (*p* = 0.59); (**c**) *MDM2* G > T gene with *T/T*, *T/G* and *G/G* polymorphisms (*p* = 0.13); (**d**) *MTHFR* C > T gene polymorphic *C/T*, *C/C* and *T/T* variants (*p* = 0.27); (**e**) SNP clustering on the average survival median (q1–q3) and mortality rates. Euclidian distances and Ward’s method were used for groups merging: (**f**) SNP’s and clinical parameters contour plot and multiple regresijon analysis. (**g**,**h**) laryngeal cancer patient survival curves according to genes SNPs, N, and HPV. Patients’ survival rates: (*p =* 0.004); (*p* ≤ 0.001).

Patients were divided into two groups, the survival rate of patients over 63 years of age was 37.5%, while the survival rate of patients up to 63 years was 66.1% (*p* = 0.03). Survival rates of patients with LSCC are associated with the spread of tumor tissue to regional lymph nodes. Lymph node injury status analysis also showed a statistically significant difference in the survival rate of N0 (66.3%), N1 (42.9%), N2 (11.1%) (*p* = 0.01) (Figure 2a). An analysis of survival rates according to HPV status did not succeed in determining a reliable relationship: the survival rate for HPV-positive patients was 54.9% and for HPV-negative it was 50.0% (*p* = 0.59) (Figure 2b). However, at the mid-point of the study (after 600 days), the patients survival difference was the highest: 57.1% of the HPV-negative patients survive in comparison with 71.4% of HPV-positive. Also, it is important to state, that the majority of patients with N2 lymph nodes status were detected as HPV-negative. The remaining 5 of 16 (31.25%) patients with metastasis in regional lymph nodes were HPV-positive.

Due to the lack of completed SNP cases of Arg/Arg and Pro/Pro, survival rate curves according to *TP53* gene Arg/Pro, Arg/Arg, and Pro/Pro SPNs cannot be calculated. In the cases of *MDM2* overall survival difference between SNP groups was not statistically significant, but in *MDM2* *T/T* cases the survival was 65.6% and *T/G* survival was 46.0% at 1631 days’ post-surgical operation (χ^2^ = 4.0, df = 2, *p* = 0.13) (Figure 2c). Regarding *MTHFR*, the overall survival difference between SNP groups also was not significant, only the *MTHFR* *T/T* survival was 80% and *C/T*, *C/C* survival was 54.0%, 43.5% at 1631 days’ post-surgical operation (χ^2^ = 2.6, df = 2, *p* = 0.27) (Figure 2d). A similar situation was shown after calculating overall survival rates in the SNP groups of *MDM4*c.1q32 A > C, *CASP8*c.-652 6N *ins/del* and *CCR5*-Δ32: any statistically significant differences were not observed after assessing survival rates.

### 3.3. Cluster Analysis of TP53, MDM2, MDM4, MTHFR, CASP8, CCR5 Genes Polymorphic Variants and Clinical-Pathological Characteristics

Cluster analysis was performed on the basis of the patients’ mortality rates and survival times median to determine which of the analyzed SNPs resulted in the best patient survival. It should be emphasized that clusterization of each SNP mostly depended on the survival time median, Q1 and Q3 (represented in Table 3 as interquartile range (IQR)). Cluster analysis identified three patient groups that contained distinct variants of SNPs. In the first cluster group (I), patient survival was 601 days and in the second group (II) the survival of patients was 522–312 days, and in the last group (III) survival of patients was 250–210 days. Table 3 and Figure 2e,f provide the results of cluster analysis. Cluster analysis of the group showed that patients with *MDM2* T/T polymorphism (cluster I) had the best survival prognosis and had the lowest mortality rates. Patients identified as *CASP8* *ins/ins*, *MTHFR* *C/C* and *MDM4* *A/A* SNP carriers (cluster III) had the worst survival probability. The highest mortality rate was in groups of patients who were identified with *MTHFR* *C/C* (55%) and *CASP8* *ins/del*, *MDM2* *T/G* (54%) SNP.

A study of contour plot and multiple regression analysis showed that patients with *MDM2* *T/T* and N0 status, had the best survival prognosis (Figure 2g). However, HPV-negative N1 and HPV-positive N0 patients with *CASP8* ins/ins variant had the poorest survival (Figure 2h).

### 3.4. Cox Model Analysis of TP53, MDM2, MDM4, MTHFR, CASP8, and CCR5 Genes Polymorphic Variants and Clinical-Pathological Characteristics

Cox proportional hazards analysis was performed to evaluate the association of age and N status, clinical stage, HPV status and SNP with LSCC survival rates. The study revealed that patients with N0 and *MDM2 T/T* had the lowest hazard ratio, and isolated hazard ratio was 0.25 (0.07–0.85). However, adjusted hazard ratio was not a significant factor (Table 4). From rezults of the study we evaluated that 90 subjects are required to achieve 0.8 power as calculated hazard ratio is 0.36 and proportion of subjects died of the disease is 0.47. Thus, the sample size of this study is not sufficient to assess N0 and *MDM2 T/T* adjusted risk.

## 4. Discussion

In this study, we analyzed patient’s survival associations with clinical parameters, HPV infection, and multiple cancer-related SNPs in 49 LSCC patients from the National Cancer Institute of Vilnius (Lithuania). By analyzing the interactions of the SNPs of the genes with other clinical parameters, such as TNM classification, we established the relationship between the presence of *MDM2*, *CASP8*, *CCR5* genes and N-region lymph nodes. The majority of patients with N2 were also identified as carriers of *MDM2* *T/G*, *CASP8* *ins/del*, and *CCR5* *wt/wt* polymorphic variants (*p* = 0.24; *p* = 0.19; *p* = 0.11). *MTHFR* *C/T* and *C/C* polymorphic variants (*p* = 0.22) were commonly found in the T4 stage primary tumor.

Meta-analysis performed by Gama R.R. et al. including 7347 cases from 179 studies showed that HPV infection was detected in 1830 (25.00%) [36]. In our study of LC patients, HPV infection was found in 42.86% (21 out of 49). For most of the patients with N2, HPV infection was not found. This may be due to the complicated availability of HPV infection to the distant lymph nodes. However, we found that polymorphic variants of the *MDM2* gene *G/G* (4 out of 28, 18.8%) and *MTHFR* gene *T/T* (4 out of 21, 19.1%)) were more common in the samples of HPV-positive and HPV-negative patients.

The associations found for other clinical features were consistent with the findings of previous studies, as age and regional metastasis in lymph nodes (N) correlated with worse prognoses. Quan F. et al. found that five clinical characteristics: laryngectomy, tumor differentiation, tumor status (T), regional N status, and clinical (TNM) stage, were correlated with patients survival [5]. We performed Cox analysis to evaluate the association of age, T stage, N status, clinical stage, HPV status, SNP with LSCC survival rates. An analysis of survival rates with HPV and N1 status did not succeed in determining a reliable relationship, but the survival rate for HPV-positive patients was >60.0% and in HPV-negative, it was <10.0% (*p* = 0.48). In the majority of patients who were identified to have lymph node status N2, infection was not found. Mallen-St Clair J. et al. found that patients with HPV associated Head and Neck Squamous Cell carcinoma (HNSCC), have an improved prognosis. The HPV status of HNSCC has been demonstrated to be a prognostic factor for overall survival, as well as progression free survival [37].

A well-known functional SNP in the tumor suppressor gene *TP53,* leads to increased longevity: in the Danish general population, homozygotes for the minor allele (*C/C*) versus homozygotes for the major allele (*G/G*) had an increase in median survival of 3 years [16]. In this study, the survival rates of the *TP53* gene polymorphism were not available to be analyzed, because of the sample homogeneity. Difference between survival groups was not significant, but *MDM2* *T/T* survival was 65.6% and *T/G* survival was 46.0% at 1631 days’ post-surgical operation (*p* = 0.13). Zhua X. et al. results indicate that homozygous *G/G* alleles of *MDM2* SNP309 may be a low-penetrant risk factor for HNSCC, and G allele may confer nasopharyngeal Canter susceptibility [26]. Data were confirmed in the Asian population: in a study of 103 patients, the *G/G* genotype of *MDM2* SNP309 was associated (*p* = 0.032) with an earlier onset of HNSCC. The average age at tumor onset was 65.6 years for *T/T*, 62.9 years for *T/G* and 56.7 years for *G/G*. The patients with the *G/G* genotype had a significantly earlier tumor onset in comparison to those with the *T/T* genotype [17,38]. Difference between survival groups was not significant, as the *MTHFR* *T/T* survival was 80% and *C/T*, *C/C* survival were 54.0%, 43.5% at 1631 days’ post-surgical operation (*p* = 0.27). Anders Q. S. et al. study showed that LSCC patients treated with chemotherapy showed an association between gene *MTHFR* C677T and survival, which was confirmed by multivariate analysis. The study demonstrated that the presence of at least one T allele decreased mortality threefold [25]. No differences were found between *MDM4* A > C, *CASP8* 652 6N *ins/del* and *CCR5*-Δ32 SNP’s in assessing survival rates. A study of contour plot and multiple regression analysis showed that patients with *MDM2* *T/T* and N0 status, had the best survival prognosis. However, HPV-negative N1 and HPV-positive N0 patients with *CASP8* *ins/ins* variant had the poorest survival. Only several similar studies have been performed. According to the Spence T. et al. study, HPV-positive and HNSCC association shows improved medical treatment response and survival rates in comparison with HPV-negative and HNSCC association [39]. The Chen WC. et al. study demonstrated that the HPV-positive LSCC showed a trend towards a better 5-year survival (100% & 85%; *p* = 0.15) and a significant improvement in the local/regional control rate (100% & 75%; *p* = 0.05), comparing to HPV-negative. Less aggressive tumor behavior and a better response to adjuvant radiotherapy/concurrent chemoradiotherapy of HPV-positive tumors were possible causes of these clinical outcomes [40]. Studies showed that additional chemotherapy for HPV-positive oropharyngeal cancer patients, may improve patients’ survival according to HPV status: HPV (and p16) positive patients were associated with longer survival compared with HPV (or p16) negative patients. Vermorken JB. et al. and Lu S. et al. study [41,42] showed that Canter immunotherapy could be applied together with chemotherapy for HNSCC patients according to HPV status, and further studies of the new biomarkers—immunological, genetic, or molecular—are needed to be initiated to provide novel targets for improvement of HPV associated HNSCC patient therapy. In addition, markers to identify HPV-positive laryngeal cancer patients with improved prognosis are emerging [43]. These insights are critical to improving our management of this rising disease and exploring effective new treatments.

However, the limitation of this study is small number of patients, included in the study. Due to these limitations the large distribution of different SNP was stated and from that we cannot get reliable significant statistical results. Probably higher number of patients with longer follow up period should be investigated in the further study.

## 5. Conclusions

In our study, contour plot and multiple regression analysis showed that patients with *MDM2 T/T* polymorphic variant and without metastasis in regional lymph nodes (N0), had the best survival prognosis. Also, analysis showed that patients with HPV-negative disease, with identified metastasis in lymph nodes (N1), and HPV-positive patients without metastasis in regional lymph nodes (N0) with CASP8 ins/ins variant had the poorest survival.

Finally, regardless of the statistically significant data, information about HPV status and the SNP of targeted SNPs can provide additional information to doctors and help to modify and individualize the patient’s treatment plan or strategy. Additionally, diagnostic and prognostic biomarkers, including HPV and SNP’s, may prove to yield strong clinical utility, and warrant further investigation and clinical validation.

## Figures and Tables

**Table 1:** Primer.

Gene	SNP	Primer	Amplification Products (bp)	Authors
***TP53***	Arg F	5’→TCCCCCTTGCCGTCCCAA→3’	141 bp	[30]
	Arg R	5’→CTGGTGCAGGGGCCACGC→3’		
	Pro F	5’→GCCAGAGGCTGCTCCCCCC→3’	177 bp	
	Pro R	5’→CGTGCAAGTCACAGACTT→3’		
***MDM2***	F	5’→TTCGGAGGTCTCCGCGGGAGTTCAG→3’	89 bp, 64 bp, 25 bp	[31]
	R	5’→TGCGATCATCCGGACCTCCCGCGTC→3’		
***MDM4***	F	5’→AAGACTAAAGAAGGCTGGGG→3’	134 bp, 111 bp, 23 bp	[32]
	R	5′→TTCAAATAATGTGGCAAGTGACC→3’		
***MTHFR***	F	5’→CCTTGAACAGGTGGAGGCCAG→3’,	294 bp, 168 bp, 126 bp	[33]
	R	5’→GCGGTGAGAGTGGGGTGGAG→3’		
***CASP8***	F	5’→AGTGAAAACTTCTCCCATGGCCTC→3’	139 bp, 291 bp, 396 bp	[34]
	R	5’→GATTGATACTGGCACAGTATACTTACC→3’		
	Ins	5’→GTAATTCTTGCTCTGCCAAGCTG→3’;		
	Del	5’→CCAAGGTCACGCAGCTAGTAAG→3’		
***CCR5***	F	5’→ACCTGCAGCTCTCATTTTCC→3’	111 bp, 79 bp	[28]
	R	5’→GCAGATGACCATGACAAGCA→3’		
***HPV***	MY09	5′→CGT-CCA-AAA-GGA-AAC-TGA-GC→3′	450 bp	[35]
	MY11	5′→GCA-CAG-GGA-CAT-AAC-AAT-GG→3′		

**Table 2:** Association of genotype distribution and clinical-pathological characteristics of laryngeal cancer patients.

		HPV Infection		*p*	Stage		*p*	N		*p*
Genotype	N (%)	+ (n = 21)	− (n = 28)		I–II (n = 9)	III–IV (n = 40)		0 (n = 33)	1–2 (n = 16)	
***TP53 Arg/Arg*** ***Arg/Pro*** ***Pro/Pro***	0 (0) 49 (100) 0 (0)	0 (0) 21 (100) 0 (0)	0 (0) 28 (100) 0 (0)	-	0 (0) 9 (100) 0 (0)	0 (0) 40 (100) 0 (0)	-	- 33 (100) -	- 16 (100) -	-
***MDM2*** ***T/T*** ***T/G*** ***G/G***	18 (36.7) 26 (53.1) 5 (10.2)	7 (33.3) 13 (61.9) 1 (4.8)	11 (37.5) 13 (43.7) 4 (18.8)	0.42	3 (33.3) 6 (66.7) 0 (0)	15 (37.5) 20 (50.0) 5 (12.5)	0.46	14 (42.4) 16 (48.5) 3 (9.1)	4 (25.0) 10 (62.5) 2 (12.5)	0.24
***MDM4*** ***A/A*** ***A/C*** ***C/C***	33 (67.3) 15 (30.6) 1 (2.1)	14 (66.7) 7 (33.3) 0 (0)	19 (67.9) 8 (28.6) 1 (3.6)	0.97	7 (77.8) 2 (22.2) 0 (0)	26 (65.0) 13 (32.5) 1 (2.5)	0.70	23 (69.7) 10 (30.3) -	10 (62.5) 5 (31.3) 1 (6.2)	0.74
***MTHFR*** ***C > T*** ***C/C*** ***C/T*** ***T/T***	20 (40.8) 24 (48.9) 5 (10.2)	8 (38.0) 9 (42.9) 4 (19.1)	12 (42.9) 15 (53.6) 1 (3.6)	0.21	2 (22.2) 7 (77.8) 0 (0)	18 (45.0) 17 (42.5) 5 (12.5)	0.14	13 (39.4) 15 (45.5) 5 (15.2)	7 (43.8) 9 (56.2) -	0.44
***CASP8*** ***Ins/ins*** ***Ins/del*** ***Del/del***	16 (32.7) 24 (49.0) 9 (18.3)	6 (28.6) 13 (61.9) 2 (9.5)	10 (35.7) 11 (39.3) 7 (25.0)	0.22	2 (22.2) 6 (66.7) 1 (11.1)	14 (35.0) 18 (45.0) 8 (20.0)	0.50	13 (39.4) 16 (48.5) 4 (12.1)	3 (18.8) 8 (50.0) 5 (31.2)	0.20
***CCR5*** ***wt/wt*** ***wt/*Δ*32*** **Δ*32/*Δ*32***	39 (79.6) 10 (20.4) 0 (0)	19 (90.5) 2 (9.5) 0 (0)	20 (71.4) 8 (28.6) 0 (0)	0.16	6 (66.7) 3 (33.3) 0 (0)	33 (82.5) 7 (17.5) 0 (0)	0.74	26 (78.8) 7 (21.2) -	13 (81.3) 3 (18.7) -	0.11
	**No = 49**				**No = 49**			**No = 49**		

**Table 3:** SNP-dependent survival rate (%) in group of patients who did not survive until the end of the study.

Cluster	I	II					III		
SNP	*MDM2 T/T*	*MDM2 T/G*	*TP53 Arg/Pro*	*CASP8 ins/del*	*MTHFR C/T*	*CCR5 wt/wt*	*MDM4 A/A*	*CASP8 ins/ins*	*MTHFR C/C*
% *	33	54	47	54	46	49	48	38	55
M ** (IQR)	601 (170)	312 (289)	353 (350)	428 (330)	522 (360.5)	369 (358)	250 (197)	210 (228)	229 (166)

* Mortality rate ** Survival median (IQR (Q2–Q1)).

**Table 4:** Results of Cox regression analysis (*p* < 0.001).

Variable	HR *	95% CI	*p*
**Multivariate analysis for single effects**			
HPV(-)/N1/*CASP8 Ins/Ins* and HPV(+)/N(0)	5.49	2.34–12.86	<0.001
*MDM2 T/T*	0.25	0.07–0.85	0.026
**Univariate analysis for combined effect**			
HPV(-)/N1/*CASP8 Ins/Ins* and HPV(+)/N(0)	4.49	1.87–10.8	0.001
*MDM2 T/T*	0.36	0.10–1.27	0.112

* Hazard ratio.
